# *Staphylococcus epidermidis *pan-genome sequence analysis reveals diversity of skin commensal and hospital infection-associated isolates

**DOI:** 10.1186/gb-2012-13-7-r64

**Published:** 2012-07-25

**Authors:** Sean Conlan, Lilia A Mijares, NISC Comparative Sequencing Program, Jesse Becker, Robert W Blakesley, Gerard G Bouffard, Shelise Brooks, Holly Coleman, Jyoti Gupta, Natalie Gurson, Morgan Park, Brian Schmidt, Pamela J Thomas, Michael Otto, Heidi H Kong, Patrick R Murray, Julia A Segre

**Affiliations:** 1 National Human Genome Research Institute, NIH, Bethesda, MD 20892, USA; 2 Department of Laboratory Medicine, NIH Clinical Center, NIH, Bethesda, MD 20892, USA; 3 Department of Medical Research and Technology, University of Maryland School of Medicine, Baltimore, MD 21201, USA; 4 NIH Intramural Sequencing Center, NIH, Rockville, MD 20852, USA; 5 Laboratory of Human Bacterial Pathogenesis, National Institute of Allergy and Infectious Diseases, NIH, Bethesda, MD 20892, USA; 6 Dermatology Branch, Center for Cancer Research, National Cancer Institute, NIH, Bethesda, MD 20892, USA

## Abstract

**Background:**

While *Staphylococcus epidermidis *is commonly isolated from healthy human skin, it is also the most frequent cause of nosocomial infections on indwelling medical devices. Despite its importance, few genome sequences existed and the most frequent hospital-associated lineage, ST2, had not been fully sequenced.

**Results:**

We cultivated 71 commensal *S. epidermidis *isolates from 15 skin sites and compared them with 28 nosocomial isolates from venous catheters and blood cultures. We produced 21 commensal and 9 nosocomial draft genomes, and annotated and compared their gene content, phylogenetic relatedness and biochemical functions. The commensal strains had an open pan-genome with 80% core genes and 20% variable genes. The variable genome was characterized by an overabundance of transposable elements, transcription factors and transporters. Biochemical diversity, as assayed by antibiotic resistance and *in vitro *biofilm formation, demonstrated the varied phenotypic consequences of this genomic diversity. The nosocomial isolates exhibited both large-scale rearrangements and single-nucleotide variation. We showed that *S. epidermidis *genomes separate into two phylogenetic groups, one consisting only of commensals. The *formate dehydrogenase *gene, present only in commensals, is a discriminatory marker between the two groups.

**Conclusions:**

Commensal skin *S. epidermidis *have an open pan-genome and show considerable diversity between isolates, even when derived from a single individual or body site. For ST2, the most common nosocomial lineage, we detect variation between three independent isolates sequenced. Finally, phylogenetic analyses revealed a previously unrecognized group of *S. epidermidis *strains characterized by reduced virulence and *formate dehydrogenase*, which we propose as a clinical molecular marker.

## Background

*Staphylococcus epidermidis *is a common human skin commensal, cultured from virtually every body surface of healthy individuals. The beneficial role of *S. epidermidis *is demonstrated by its ability to inhibit colonization by the pathogenic *Staphylococcus aureus *[1]. While *S. epidermidis *is a commensal on the skin, if it breaches the skin surface and enters the bloodstream, it is considered a pathogen. *S. aureus *and coagulase negative *Staphylococcus*, including *S. epidermidis*, comprise 30% of hospital-acquired infections [2], associated with an estimated $2 billion annually in treatment costs [3]. *S. epidermidis *forms biofilms on medical devices, such as contact lenses, catheters, and prosthetic heart valves. The detachment of bacterial cells from biofilms on these devices can lead to bacteremia, with increased morbidity and potential mortality [4]. In clinical settings, *Staphylococcal *species are frequently resistant to antibiotics, particularly to penicillinase-resistant penicillins (for example, methicillin, oxacillin, nafcillin), constraining treatment options. *S. epidermidis *is also suspected to be a source of genetic diversity for *S. aureus *to acquire genes enabling better adherence to skin cells [5]. Methicillin-resistant *S. aureus *(MRSA) is considered a re-emerging pathogen, showing increased drug resistance and causing an estimated 18,650 deaths per year in the US [6]. The contrasting roles of *S. epidermidis *in both health and disease make it an important and central player in the human microbiome.

Hospital patients are typically monitored for sepsis from a blood sample. However, commensal staphylococci from the skin can contaminate venipuncture cultures, leading to false positives. This complicates the decision of whether to treat with antibiotics, remove the medical device, or wait-and-see, any of which can extend the patient's hospital stay [4]. One approach to predicting the invasiveness of a strain is the use of marker genes. For instance, the *ica *operon, contributing to biofilm formation, has been proposed as a marker for invasiveness [7]. The IS256 insertion sequence has also been shown to be associated with biofilm formation and resistance to aminoglycosides [8] and has been proposed as a marker for invasive strains [9]. Despite the statistical significance of these markers, Rohde and colleagues [10] have shown that existing marker genes are not sufficient for discriminating invasive strains in a clinical setting.

While marker-based assays are important tools for epidemiological studies, a deeper understanding of *S. epidermidis *is required for foundational studies on population structure [10]. Standard microbiological typing methods such as pulsed field gel electrophoresis and multi-locus sequence typing are used to track the spread of strains and measure clonality within patient populations [11], hospitals [12] and geographic areas [13], but are inherently low resolution methods. Microarrays have been used for genome-wide analyses and have successfully identified putative virulence determinants in *S. epidermidis *strains [14]. New technologies, including optical mapping, which produce ordered restriction enzyme maps, will provide increased physical resolution of strains [15]. Recently, high-throughput sequencing and SNP analysis were used to perform a detailed study of the spread and evolution of MRSA clones [16], demonstrating the power of direct sequencing to study bacterial population dynamics. To date, genome-wide studies on *S. epidermidis *were hampered by the fact that only two complete reference genomes existed, ATCC12228 [17] and RP62A [18]. Based on the limited number of reference genomes, it was unclear how much sequence diversity existed among *S. epidermidis *strains.

The pan-genome [19], or collection of genes found among members of a species, is a useful framework for describing genomic diversity within a taxon. For example, Tettelin and colleagues [20] characterized the pan-genome of *Streptococcus agalactiae*, an important pathogen for newborn infants. Based on the analysis of publicly available genomes as well as genomes generated specifically for their study, they found that approximately 20% of an individual *S. agalactiae *genome is made up of genes that are only partially shared with other strains, including unique genes. Furthermore, they found that sequencing additional genomes was predicted to increase the size of the pan-genome, suggesting that *S. agalactiae *has an open genome. This type of analysis has been used to characterize a number of bacterial species [21] as well as genera [22] and has been used to estimate the pan-genome size of all bacteria [23].

To explore the genomic variability of commensal and nosocomial *S. epidermidis *isolates, we produced draft genome sequences for 30 isolates selected from our extensive strain collection of commensals and pathogens at the NIH Clinical Center. Comparative genomics was used to characterize the pan-genome and deduce the phylogenetic relationship between strains.

## Results and discussion

### Genomic variability of commensal *S. epidermidis*

**Figure 1:**
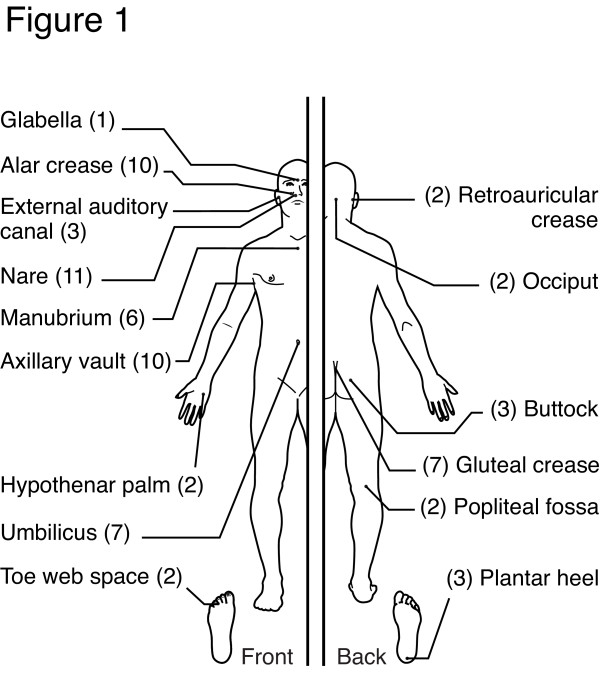
**Body sites analyzed**. A collection of 71 *S. epidermidis *isolates from 7 healthy volunteers across 15 body sites were selected for characterization (number of isolates from site in parenthesis).

**Figure 2:**
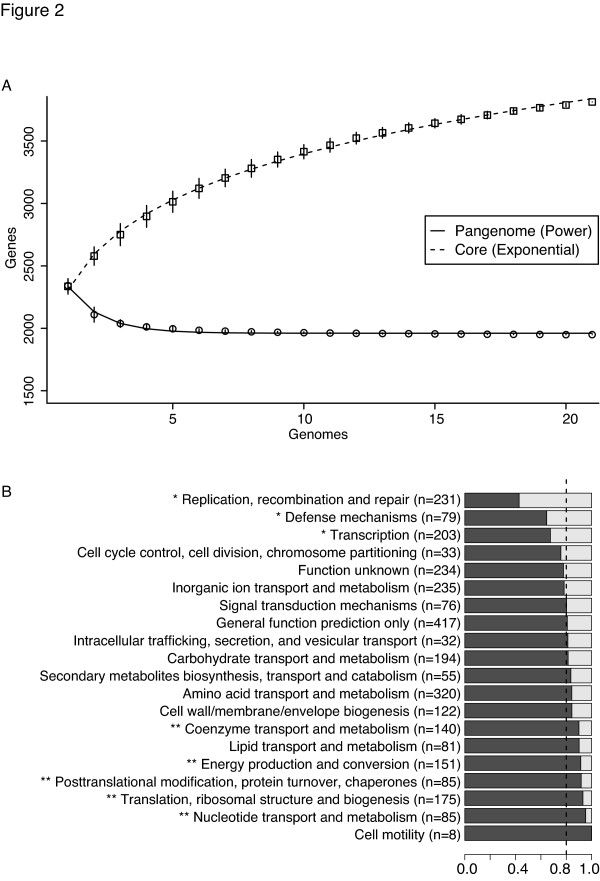
**Commensal *S. epidermidis *has an open pan-genome. **Pan-genome size of *S. epidermidis*. **(a) **Gene accumulation curves for pan-genome (squares) and core-genome (circles) as a function of genomes sequenced (*N*). Error bars are ±1 standard deviation for the 100 simulations. Core gene data are fit by: *y *= *a·exp^-N/b ^+ c*. Pan-genome data are fit by: *y *= *a·N^b ^+ c*. **(b) **Partitioning of core and variable genes for each COG. COGs significantly enriched (*P *< 0.05, Fisher exact test) in variable or core genes are marked with single and double asterisks, respectively. The dashed line indicates the expected proportions.

As part of our characterization of the human skin microbiome from healthy volunteers (HVs) [24], we cultured >800 individual bacterial strains. Based on morphologic characterization, approximately 80% of those strains were classified as the genus *Staphylococcus*. We selected 71 *S. epidermidis *isolates from 15 body sites of seven HVs (Figure 1) to further characterize and identify representative strains for sequencing. The identity of these *S. epidermidis *strains was confirmed by 16S rRNA gene sequencing and ribosomal protein matrix-assisted laser desorption/ionization time of flight (MALDI-TOF) mass spectrometry. In addition, we employed Rep-PCR [25] to investigate the genome architecture of these strains and used these data to select 21 strains for whole genome sequencing (Additional file 1). The 21 draft genomes (including plasmids) were 2.5 ± 0.1 Mb in size and 32% GC on average. Each genome had 2,436 ± 79 predicted protein coding genes. Predicted genes were clustered into orthologous groups, resulting in 3,750 gene clusters and 540 singletons. Gene accumulation curves [20] (Figure 2a) showed that the core genome size fits an exponential decay curve that plateaus at 1,960 genes while the pan-genome data fit a power law curve. The *S. epidermidis *genome, while relatively constant in size (<5% variance), was 80% core genes and 20% variable genes that were drawn from a larger pool, indicating an open pan-genome where each genome sequence added a number of new genes.

The function of the genes within the variable genome was investigated by assigning all gene clusters to clusters of orthologous groups (COG) categories [26]. Three of the 20 COG categories were significantly enriched in the variable genome by Fisher's exact test (*P *< 0.05): (1) replication, recombination and repair; (2) transcriptional regulators; and (3) defense mechanisms (Figure 2b). Enrichment of these classes was driven by diversity in mobile genetic elements (recombinase and integrase genes), transcriptional regulators, and ABC-type multidrug transporters, respectively. In addition, 40% of the genes in each genome were not assigned a COG function, reflecting novel gene clusters as well as limitations in COG classification.

### Genomic variability of nosocomial *S. epidermidis*

**Figure 3:**
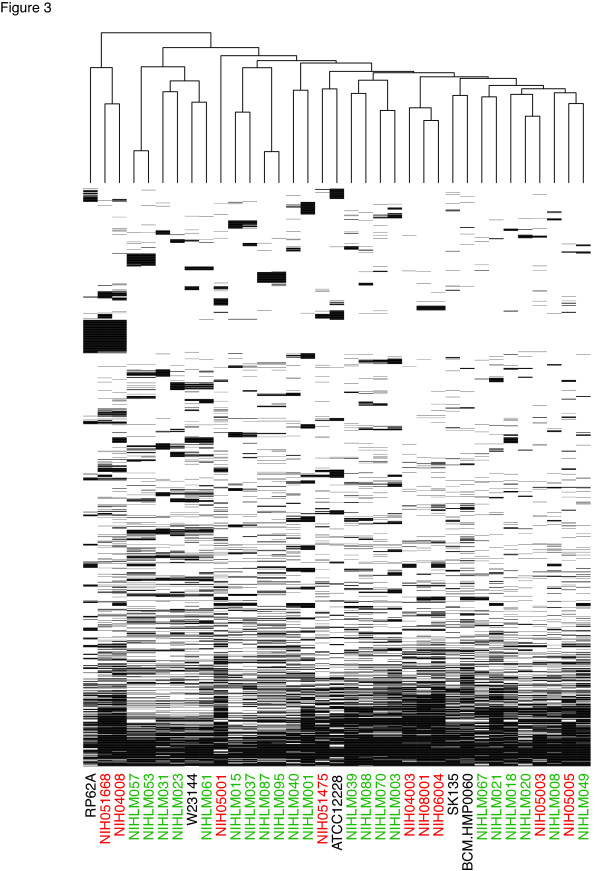
***S. epidermidis *variable genome is dominated by genes shared by few strains. **Only the variable genome is shown. Gene clusters present in all genomes or present in only a single genome are omitted. The presence of a gene (and its paralogs) is indicated by a black bar. Nosocomial and commensal isolate names are colored red and green, respectively. The dendrogram was generated using complete linkage clustering of the presence/absence data.

To investigate if nosocomial (hospital-acquired) *S. epidermidis *strains share the same genetic diversity as commensal strains, we sequenced nine nosocomial isolates from the NIH Clinical Center, seven from catheter-associated infections, one from joint fluid and one from lung biopsy tissue (Additional file 2). In clinical epidemiological studies, multi-locus sequence typing is commonly used to classify sub-types based on exact sequence matches at seven housekeeping genes [27]. All nine nosocomial isolates belong to clonal complex 2 (CC2), which comprises 74% of hospital-acquired infections worldwide and contains the dominant sequence type 2 (ST2) [28]. The isolates in this study include important CC2 founders and subfounders, as defined by Miragaia and colleagues, including three ST2 strains, two ST5 strains and one ST89 strain. To interrogate the larger *S. epidermidis *core and pan-genome, gene predictions were generated for these nosocomial isolate genomes, for three draft *S. epidermidis *genomes generated as part of the Human Microbiome Project (HMP) and for the two complete *S. epidermidis *reference genomes, yielding a total of 35 genomes (Figure 3).

**Figure 4:**
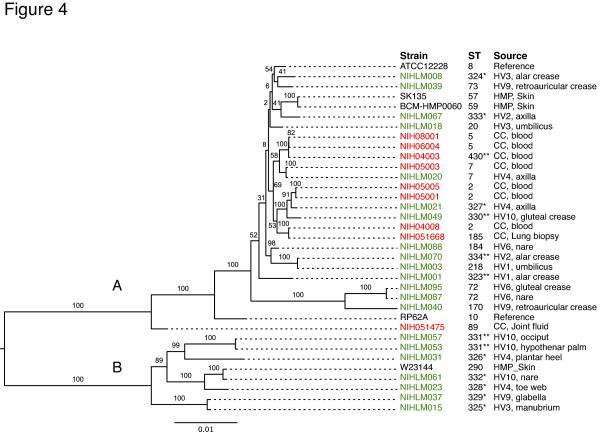
***S. epidermidis *taxon is made up of two groups. **Maximum likelihood phylogenetic tree of *S. epidermidis *based on four-fold degenerate positions of genes in the core genome. The table shows the sequence type (ST) from multi-locus sequence typing. New sequence types and sequence types with a new allele are indicated by single and double asterisks, respectively. The RP62A and ATCC12228 strains are reference strains (NC_002976, NC_004461). Isolates SK135, BCM-HMP0060 and W23144 were sequenced as part of the Human Microbiome Project Reference Genome catalog. Nosocomial and commensal isolate names are colored red and green, respectively. Bootstrap values are shown on each branch. Outgroup (*S. aureus*) not shown.

Phylogenetic relationships between the set of 35 isolates (21 commensal, 9 nosocomial, 3 HMP and 2 reference) were assessed using a maximum-likelihood tree built from 4-fold degenerate codon positions of core genes (Figure 4). The USA300 *S. aureus *TCH1516 genome was used as the outgroup to root the tree but is not shown in Figure 4. Two of the three ST2 nosocomial isolates sequenced in this study, NIH05001 and NIH05005, were near identical at the nucleotide level for all core genes, but optical mapping showed seven insertions (2 to 40 kb) and a number of inversions, translocations and duplications between these strains (Additional file 3). The third ST2 isolate, NIH04008, differed at the nucleotide level (96% identity across neutrally evolving positions) from the other ST2 isolates and contained a number of insertions, including the SP-beta prophage. Of the six other non-ST2 nosocomial isolates, the lung biopsy isolate NIH051668 was >99% identical to NIH4008 across neutrally evolving positions of core genes. Three of the catheter-derived isolates (NIH08001, NIH06004, NIH04003) were similarly closely related phylogenetically across core genes. Interestingly, NIHLM020 and NIH05003 shared the same degree of phylogenetic relatedness as above pairs of nosocomial isolates despite coming from commensal and nosocomial isolation sources, respectively. Finally, NIH051475, which was isolated from joint fluid, is the most distantly related of the nosocomial isolates in this study.

### Phylogenetic relationship of *S. epidermidis *commensal and nosocomial genomes

Strikingly, the 35 *S. epidermidis *isolates formed two distinct groups, called A and B (Figure 4) with excellent bootstrap support. All of the nosocomial isolates mapped to the upper group A in the phylogenetic tree. In contrast, the commensals were distributed between groups A and B. The 24 commensal isolates fall into 22 different sequence types, including many new sequence types (12/22), made up of new combinations of alleles as well as novel alleles. When overlayed on the phylogenetic tree, isolates with new sequence types were disproportionately found in the group B (7/8 isolates) compared to group A (7/27 isolates) (Figure 4; *P *= 0.01). Furthermore, eBurst [29] analysis shows that the majority of group A commensal isolates belong to the CC2 lineage while none of the sequence types in group B are connected to the CC2 lineage.

**Figure 5:**
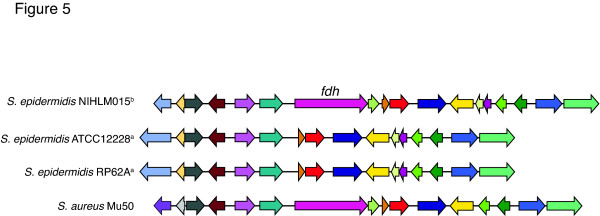
***fdh *gene neighborhood shows co-linearity across staphylococci**. The gene neighborhood of *fdh*. Strains from group A and B are marked with superscript 'a' and 'b', respectively. Genes with conserved function are the same color. Genome alignments were generated using RAST.

Only ten genes strictly differentiated group A and B isolates, with nine encoding short proteins of unknown function. The exception was the formate dehydrogenase (*fdh*) gene, encoding a 983 residue protein, which was found only in the group B isolates. The regions flanking the *fdh *gene are 98% identical across all sequenced *S. epidermidis *isolates and are contiguous in group A isolates (Figure 5). The *S. epidermidis fdh *gene is 80% identical to an orthologous *S. aureus *gene with conserved co-linearity, suggesting the *fdh *gene was lost by group A isolates.

Since the group B isolates are solely commensal in origin, phylogenetically distinct and represent largely novel sequence types, we hypothesized that they could represent an undersampled lineage of *S. epidermidis*, possibly with reduced virulence. We tested this by assaying marker genes in a larger set of 71 commensal strains and 46 nosocomial strains. First, we used PCR to determine the presence of three markers of invasive strains as demonstrated by Rohde *et al*. [10]: *icaA*, *mecA *and IS256. As in Rohde *et al*., we found these three markers to be significantly associated with nosocomial strains (*P *< 0.01). In slight contrast to Rohde's results, we found *icaA*, *mecA *and IS256 at higher rates in our commensal isolates, which reduced their discriminatory power in our isolates (Table 1). Next, we typed our nosocomial and commensal isolates using the *fdh *gene as a marker and found it in approximately 23% of commensal strains (16/71) but only approximately 4% of nosocomial strains (3/46) (*P *= 0.02). Interestingly, the three *fdh *positive nosocomial isolates had few virulence markers, as determined by the absence of *icaA *or *mecA *genes and two of three with a weak IS256 band. This could suggest that these three isolates, while classified as nosocomials from a blood draw, may actually represent commensal contaminants from the venipuncture. In contrast to traditional nosocomial markers, *fdh *is a commensal-associated marker with discriminatory potential.

**Table 1:** The *fdh *gene is a marker of commensal strains

	Prevalence (%)	
	
	Commensal (n = 71)	Nosocomial^a ^(n = 46)
*Fdh*	22.5% (16)	6.5% (3)
*icaA*	33.8% (24)	63.0% (29)
*mecA*	15.5% (11)	80.4% (37)
IS256	4.2% (3)	47.8% (22)

The prevalence of each gene was surveyed using PCR as described in the Materials and methods. Of 46 nosocomial isolates, 20 were isolated from positive blood culture, 23 from IV catheter and 3 from matched blood and IV catheter.

### Discriminating commensal and nosocomial isolates

Group A isolates were a mix of nosocomial and commensal isolates, raising the question of whether differences existed in gene content between the 9 nosocomial and 14 commensal group A strains. While no gene clusters discriminated perfectly, 21 clusters were enriched in genes from nosocomial group A genomes, including nine in the SCC*mec *cassette, the IS256 element and the *aacA *antibiotic resistance gene. In addition to genes encoding hypothetical proteins, three orthologous gene clusters (RP62A genes SERP0245 to SERP0247) were found in nine nosocomials and only 3 of 14 commensal isolates. These genes encode a putative transport system and transcriptional regulator, possibly associated with antibiotic resistance. As such, while the nosocomial and commensal isolates clustered together phylogenetically, there appeared to be gene content adaptations to hospital life.

**Figure 6:**
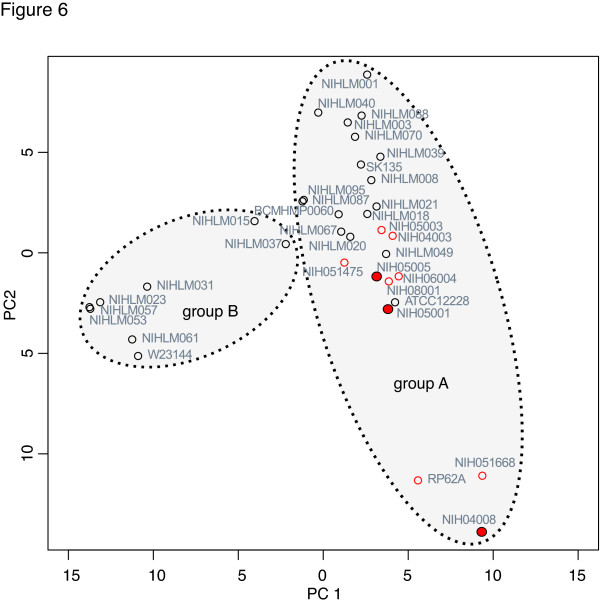
**Principal components anaylsis of ortholog clustering**. The orthologous gene cluster data for 35 genomes was analyzed using principal components analysis. Nosocomial isolates are shown as red circles and ST2 nosocomial isolates are filled red circles. Commensal isolates are black circles. The dashed ovals indicate the phylogenetic groups described in Figure 4 but are not statistically defined. Percentages of variance explained by PC1 and PC2 are 13.8% and 9.1%, respectively.

We applied two additional analyses to our data to identify patterns differentiating commensal from nosocomial isolates. Principal components analysis was used to visualize the ortholog abundance data shown in Figure 3. In agreement with the phylogenetic tree, group A and B isolates occupied non-overlapping regions of the graph (Figure 6). The group A isolates clustered into an ellipse with isolates forming a gradient from commensal to nosocomial. This clustering suggests that *S. epidermidis *isolates exist on a continuum between benign and pathogenic lifestyles. Patterns in these data were further investigated by using a random forest classifier to separate nosocomial and commensal isolates based on orthologous gene clusters. The random forest was able to classify isolates as commensal or nosocomial with an error rate of 11%. When compared to the principal components analysis data, misclassified isolates were always located in the middle of the cluster where some mixing of commensal and nosocomial isolates is seen. Taken together, these data show that *S. epidermidis *exists on a continuum of pathogenicity that integrates both phylogeny and gene content.

### *S. epidermidis *biochemical properties: biofilm formation and antibiotic resistance

**Figure 7:**
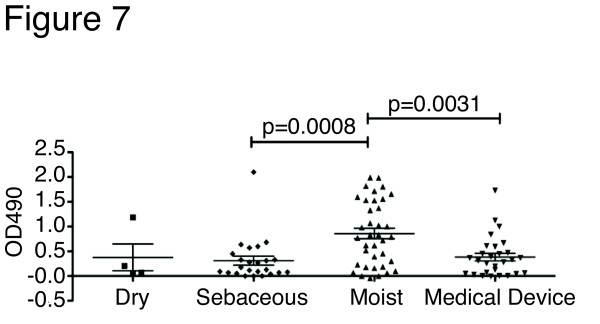
***in vitro *biofilm formation is associated with isolation source**. *in vitro *biofilm formation on polystyrene surface of 71 commensal and 28 medical device (nosocomial) *S. epidermidis *isolates. Commensal isolates are subdivided by the skin type (dry, sebaceous and moist) as defined in Grice *et al*. [24]. Each point represents the average of four replicates. Statistical significance was calculated using Mann-Whitney, two-tailed.

*S. epidermidis *harbors few classical virulence determinants [3], but genes involved in biofilm formation and antibiotic resistance contribute to the persistence of clinical infection [30]. Biofilm formation is assayed *in vitro *by measuring the bacteria's ability to form a surface-attached aggregation. By this assay, we found that biofilm formation was widespread amongst both commensal and nosocomial *S. epidermidis *isolates. The *ica *genes, encoding biofilm-associated genes for poly-*N*-acetylglucosamine synthesis, were found in only 60% of the biofilm-forming commensal isolates, in agreement with previous studies [31]. This suggests genetic heterogeneity underlying the mechanism by which *S. epidermidis *forms a biofilm. Interestingly, biofilm formation was associated with the site of origin; isolates from moist skin sites demonstrated an enhanced ability to form biofilms compared to those from oily sites (Figure 7). In fact, commensal isolates from moist skin sites have a greater ability to form biofilms *in vitro *than even those isolated from medical devices.

Antibiotic resistance is common among *S. epidermidis *isolates and often limits treatment options. Isolates from medical devices were largely resistant to methicillin (21/28). Among the commensal isolates examined in this study, methicillin resistance was confined to isolates from HV10 (9/32). Commensal isolates were less likely to be resistant to trimethoprim-sulfamethoxazole (4/32) or clindamycin (2/32) than nosocomial isolates, which were resistant to each drug in >50% of isolates tested. First-line antibiotic therapy for catheter-related bloodstream infections is vancomycin. None of the isolates tested were resistant to linezolid, quinupristin-dalfopristin or vancomycin, regardless of isolation source.

A defining feature of methicillin-resistant *S. epidermidis *(MRSE) is the presence of the SCC*mec *cassette; a heterogenous sequence defined by combinations of *mec *gene complexes, cassette recombinases and accessory genes. In agreement with the results of Li and colleagues [12], the ST2 nosocomial isolates have a type III mec cassette. In fact, all of the nosocomial isolates with an SCC*mec *cassette (8/9) were either type III or type IV, as found in previous studies of CC2 strains [28]. Both MRSE and methicillin-sensitive *S. epidermidis *(MSSE) isolates were cultured from HV10 across multiple skin sites. Comparing MRSE (9/15) and MSSE (6/15) isolates from HV10, the ability to form biofilms did not correlate with resistance to methicillin, a finding that was not surprising given that biofilm-formation alone confers a degree of antibiotic resistance. Of the sequenced MRSE commensal isolate genomes, NIHLM061 and NIHLM053/057 had type IV and V cassettes, respectively. NIHLM049 had a *mecA *gene and partial *mecR1 *gene but no detectable cassette recombinase genes. These multiple unique SCC*mec *cassettes demonstrated the independent acquisition, selection and persistence of multiple MSSE and MRSE isolates within a single individual.

### *S. epidermidis *plasmids and CRISPR

Mechanisms of resistance to other antibiotics were generally explained by the presence of common resistance genes. A number of these genes are likely carried on plasmids, including pUB110 (bleomycin/kanamycin), pSE-12228-01 (tetracycline), and pKH19 (erythromycin). Some resistance phenotypes could not be immediately accounted for by the gene catalog and may be explained by novel genes [32]. For instance, tetracycline resistance is explained by the presence of the pSE12228-01 plasmid in two isolates but three other isolates (NIHLM053/057/061) had no obvious tetracycline resistance genes, suggesting an unidentified gene product may be responsible. For the 30 genomes sequenced in this study, nucleotide homology searches produced strong evidence of 13 different plasmids (free or integrated) in 17 isolates.

In addition, we detected five strains with clustered, regularly interspaced, short, palindromic repeat (CRISPR) loci, which confer sequence-specific immunity to bacteriophage and restrict the spread of conjugative plasmids [33]. CRISPR regions in these five strains contained two to three loci with three to ten spacer regions each. Approximately 90% of the unique spacers identified in this study did not match targets in the public sequence database, pointing to a large pool of unsequenced staphylococcal phage and plasmids, similar to recently described streptococcal CRISPR diversity [34]. One exception was a nine spacer CRISPR in NIH06004, with five spacers matching plasmid borne proteins, including *pre*, *traE *and *rep*.

## Conclusions

This study presents the largest analysis of *S. epidermidis *genome sequences to date, and includes both commensal and nosocomial isolates to examine the full pan-genome. Our analysis demonstrates that *S. epidermidis *has a relatively compact genome with a fixed size of approximately 2.5 Mb, and yet as much as 20% of this genome is in flux, exchanging with a large pool of genes. These values are in agreement with what was reported by Tettelin and colleagues [20] for the *S. agalactiae *pan-genome and led us to similar conclusions about the importance of sequencing many isolates from a species. Recently, it was shown for *Salmonella enterica *that core gene variation could be used to construct high-resolution phylogenetic trees, similar to what is shown in Figure 4, that reveal important details not seen by traditional multi-locus sequence typing (MLST) [35]. In addition, they identified a subset of core genes with high sequence variability that appear to be under selection. We also find a small number of core genes with higher than average variability (as measured by average entropy across aligned columns). The etiology of this variability is unclear, but, interestingly, many of the core genes were syntenic gene clusters, including SERP2041-2043 and *ureFGD *(gene names from the RP62A genome; NC_002976). In a detailed study of the pan-genome of the gut microbe *Methanobrevibacter smithii*, Hansen and colleagues [36] showed that principal components analysis of orthologous genes was able to cluster strains by family of origin. We did not observe a clustering by individual, but this may be related to the ecological heterogeneity of the skin environment (moist, oily, dry) compared to the gut.

Analysis of the phylogenetic tree based on the 35 genomes in this study showed that *S. epidermidis *strains clustered into two distinct groups A and B, differentiated by the *fdh *gene (group B). Group B represents a lineage with reduced virulence, as nosocomial genomes are found only within group A. Moreover, in a larger replication set of 117 *S. epidermidis *isolates, the *fdh *gene had discriminatory power as largely absent in nosocomials. While clinical markers have traditionally focused on what 'is' present in nosocomials versus commensals, the *fdh *gene has the potential to identify possible contaminants in the blood culture from the venipuncture procedure.

Within group A, nosocomial and commensal strains are intermingled phylogenetically, but nosocomials are still enriched in markers traditionally associated with antibiotic resistance. The observation that isolates exist on a gradient of pathogenicity is further supported by principal components analysis of gene content that shows separation based on isolation source (hospital infections versus healthy volunteers). However, strains from both healthy individuals and hospital-associated infections can have nearly identical genomes (for ecample, NIHLM020 from a healthy volunteer's axilla and NIH05003 from a leukemia patient's catheter) indicating that opportunity and environment clearly contribute to hospital-acquired infections. Furthermore, a single individual can carry many *S. epidermidis *strains with differing antibiotic resistance profiles, capacities to form biofilms and overall gene compositions. These data, combined with the difficulty assigning isolates as strictly commensal or nosocomial, highlights the challenges associated with identifying the genetic determinants of virulence.

The ST2 lineage is the most common hospital-acquired strain. However, it is unclear why this particular lineage is dominant. The three ST2 strains sequenced in this study, the first sequences of this dominant sequence type, are similar but not nearly as identical as MRSA USA300 genomes. The only genes we found specifically associated with ST2 strains were part of the type III SCC*mec *element, which suggested additional roles for this cassette in *S. epidermidis *virulence. For instance, type III cassettes carry a phenol soluble modulin *psm-mec *that has been shown to affect the virulence of *S. aureus *[37]. While the acquisition of antibiotic resistance genes and other defense mechanisms may predispose a strain to a pathogenic lifestyle, it may also be a consequence of exposure to a hospital environment.

Metagenomics is one of the ultimate goals of the HMP. While the initial metagenomic sequencing projects relied upon alignment to a limited set of 'reference' genomes, here we clearly show that *S. epidermidis *should be viewed as a pan-genome to empower full metagenomic studies. While complicating a one-to-one assignment of genes to a species, this pan-genomic understanding opens the window to future studies, such as defining the composition of *S. epidermidis *strains on catheter-derived biofilms and their roles in establishment of infection. This representative catalog of commensal and pathogenic staphylococci from the skin will also empower quantitative assessments of changes in staphylococcal burden during disease exacerbation, such as chronic relapsing atopic dermatitis or MRSA infection. Finally, these results pose the evolutionary question of how and why ST2, the most common nosocomial strain, has been optimized for a pathogenic lifestyle, given the large number of gene combinations available in the *S. epidermidis *pan-genome.

## Materials and methods

### *S. epidermidis *isolates

Commensal isolates were obtained from swabs of healthy volunteers as described previously [24], classified by 16S rRNA gene sequencing and verified with ribosomal protein signature provided by MALDI-TOF mass spectrometry. The DiversiLab Staphylococcus kit for DNA fingerprinting (Bacterial Barcodes Inc., Athens, GA, USA) was used to fingerprint 71 isolates; detection and cluster analysis of fingerprint patterns was performed with DiversiLab software version 3.3. Of 71 isolates, 21 were selected for further characterization based on observed Rep-PCR profiles. Isolates were grown on sheep blood agar and passed three times to confirm homogeneity of the strain. The strains were maintained as frozen glycerol stocks at -80°C. Medical device-associated strains were collected between 2004 and 2010 at the NIH Department of Laboratory Medicine. Genomic DNA was prepared using MoBio Laboratories UltraClean Microbial DNA kit (Mo Bio Laboratories, Inc., Carlsbad, CA, USA) according to manufacturer instructions. DNA was quantified prior to sequencing using the Quant-iT dsDNA BR assay (Invitrogen, Grand Island, NY, USA). All strains sequenced in this study are available from BEI Resources under BEI numbers HM-896 to HM-925.

### Functional assays: antibiotic susceptibility and biofilm formation

MicroScan (Dade Behring, CA, USA) dried MIC/Combo Gram Positive panels were used to evaluate biochemical properties and antibiotic sensitivities based on manufacturer's protocol. Biofilm assays were performed according to Vuong *et al*. [38]. Briefly, *S. epidermidis *isolates from a frozen stock were used to inoculate 1 ml of trypticase soy broth, grown overnight and then a 1:100 dilution was prepared in 0.5% glucose trypticase soy broth. Wells of a microtiter polystyrene plate (BD Falcon, Franklin Lakes, NJ, USA) were inoculated in triplicate. After incubation for 24 h at 37°C, the plates were washed twice with double distilled water and stained with 0.1% safranin for 1 minute. Excess safranin was removed by blotting and air-drying. Absorbance was read at 490 nm using a SpectraMax 384 (Molecular Devices, Sunnyvale, CA, USA) with SpectraMaxPro software.

### Molecular typing

MLST was carried out using sub-sequences for seven housekeeping genes [27] extracted from the draft genomes. Any allele that did not match an existing allele in the *S. epidermidis *MLST database [39] was verified by manual examination of the sequence assembly for coverage and quality and Sanger sequencing. New sequence types and alleles were submitted for inclusion in the *S. epidermidis *MLST database. PCR assays for *icaA*, *mecA *and IS256 were performed as described by Rhode *et al*. [10]. The *fdh *gene was detected by PCR by using the following primers: forward, 5'-ATA ATG GTG ATA TTC ATT CG; reverse, 5'-CCG TAT TAG TAA AAG TTC CA. Universal primers against the 16S gene were used as a positive control. SCCmec cassette types were determined by comparing all contigs to a database of SCCmec protein sequences derived from the following GenBank accessions: AB033763, D86934, AB037671, AB063172, AB121219, AF411935, FJ390057. While SCCmec cassettes cannot be definitively subtyped from draft genomes due to fragmentation by multiple transposable elements, a preliminary classification was made based on *mec *gene complex, *ccr *gene complex and characteristic joining regions. Types were assigned based on the type descriptions from the International Working Group on the Classification of Staphylococcal Cassette Chromosome Elements (IWG-SCC) [40].

### Genome sequencing and annotation

Isolates were sequenced on a Roche 454FLX Ti instrument (Roche Diagnostics GmbH, Mannheim, Germany) by the NIH Intramural Sequencing Center. All isolates were initially sequenced using unidirectional fragment reads; 12 genomes were supplemented with paired-end reads (3 kb insert size). Genomes were assembled using the Roche gsAssembler v2.3 (091027_1459). All genome assemblies exceeded the provisional assembly metrics set forth by the HMP [41]. Specifically, >90% of the genome included in the contigs, >90% of bases have >5× read coverage, >5 kb contig N50, >20 kb scaffold N50 for genomes with paired ends, >5 kb average contig length, >90% of core genes found. Optical maps of selected strains were generated by OpGen (Gaithersburg, MD, USA) and used to identify large-scale changes in genome architecture. Glimmer v.3.02 [42] was used to predict protein-coding genes. Orthologous protein clusters were generated using OrthoMCL [43]. Analysis using TBLASTN indicated that relatively few genes were absent due to errors in gene calling or pseudogene formation. Unclustered genes were filtered as described in Lefébure and Stanhope [22]. Briefly, genes were retained as singletons only if they were at least 50 amino acids long and did not have homology to other proteins by BLAST (E-value <1e-10). Final annotation of each genome was generated using the National Center for Biotechnology Information's (NCBI's) Prokaryotic Genomes Automatic Annotation Pipeline (PGAAP) and further processed by the HMP annotation working group pipeline, which ensures uniform gene product naming. Assemblies and raw sequencing data (SRA) are available from GenBank under BioProject 62343.

COGs [26] were assigned to proteins by first aligning each putative protein sequence against a BLAST database of COG sequences, generated from a file downloaded from the NCBI [44]. A protein was annotated as belonging to a given COG based on the criteria described in Tatusov *et al*. [26]. Specifically, a protein was assigned to a COG if the best hit in at least two of the COG genomes is annotated to the given COG. CRISPR loci were detected using CRISPRfinder [45] and confirmed by the presence of CRISPR-associated (*cas*) genes on the same contig. Putative extrachromosomal elements (for example, plasmids) were identified by inspection of paired-end data and BLASTN alignment to the complete plasmid database from NCBI. Antibiotic resistance genes were identified using ARDB [46].

The phylogenetic tree was generated from four-fold degenerate positions in a subset of core gene sequences. Core gene clusters without paralogs were aligned using Muscle v.3.7 [47] and ambiguous alignments were removed. Four-fold degenerate codon positions were extracted from the resulting alignments, filtered to remove positions with low quality bases and concatenated. A phylogenetic tree was built using PhyML v.2.4.4 [48]. Single nucleotide polymorphisms were identified in draft genomes by aligning them to the ATCC12228 genome using ABACAS [49] and then filtering the resultant SNP calls to remove misalignments, homopolymer errors and low quality calls [50].

### Statistical tests

The significance of core gene abundance in COG categories was examined using Fisher's exact test as implemented in R (v.2.10.0). Principal components analysis was performed using the *prcomp *function in R. Random forest classification was performed using the *randomForest *package in R. The P-test [51], as implemented in Unifrac suite was used to determine if environment labels were significantly associated with the structure of the phylogenetic tree of isolates.

### Data availability

This whole-genome shotgun project has been deposited at DDBJ/EMBL/GenBank under BioProject 62343. All genomic sequence data links are individually deposited under the accession numbers: AKGI000000000, AKGJ00000000, AKGK00000000, AKGL00000000, AKGM00000000 AKGN00000000, AKGO00000000, AKGP00000000, AKGQ00000000, AKGR00000000, AKGS00000000, AKGT00000000, AKGU00000000, AKGV00000000, AKGW00000000, AKGX00000000, AKGY00000000, AKGZ00000000, AKHA00000000, AKHB00000000, AKHC00000000, AKHD00000000, AKHE00000000, AKHF00000000, AKHG00000000, AKHH00000000, AKHI00000000, AKHJ00000000, AKHK00000000, AKHL00000000. The version described in this paper is the first version.

Short Read Archive study numbers are: SRP012973, SRP012974, SRP012975, SRP012977, SRP013026, SRP013029, SRP013030, SRP013031, SRP013032, SRP013033, SRP013034, SRP013035, SRP013036, SRP013037, SRP013038, SRP013041, SRP013042, SRP013043, SRP013044, SRP013045, SRP013046, SRP013047, SRP013048, SRP013049, SRP013050, SRP013107, SRP013108, SRP013149, SRP013165, SRP013189.

## Abbreviations

CC2: clonal complex 2; CRISPR: clustered regularly interspaced short palindromic repeats; COG: clusters of orthologous groups; *fdh*: formate dehydrogenase; HMP: Human Microbiome Project; MALDI-TOF: matrix-assisted laser desorption/ionization time of flight; MLST: multi-locus sequence typing; MRSA: methicillin resistant *S. aureus*; MRSE: methicillin-resistant *S. epidermidis*; MSSE: methicillin-sensitive *S. epidermidis*; NCBI: National Center for Biotechnology Information; PCR: polymerase chain reaction; SNP: single nucleotide polymorphism; ST: sequence type.

## Authors' contributions

SC carried out the genomic analysis studies, and drafted the manuscript. LAM carried out the microbiologic isolation and characterization of the isolates and drafted the manuscript. NISC, JB, RWB, GBB, SB, HC, JG, NG, MP, BS, and PJT prepared DNA libraries, performed sequencing and participated in sequence alignment and analysis. MO designed biofilm assays. HHK, PRM and JAS designed and coordinated studies. All authors read and approved the final manuscript.

## Competing interests

The authors declare that they have no competing interests.

## Supplementary Material

Additional file 1 **Genome assembly metrics**. The Contig N50 indicates that 50% of the bases are in contigs of this size or larger. Average coverage is the average number of reads contributing to a given position in the contig. Click here for file

Additional file 2 **Strain metadata**. Subject metadata are presented for each strain sequenced in this study. The age column indicates the age of the subject at the time of strain isolation. The subject race/ethnicity is indicated as white, black or hispanic. For nosocomial isolates, a condensed patient history is provided leading up to strain isolation. Click here for file

Additional file 3 **Optical mapping of ST2 isolates NIH05001 and NIH05005**. Optical maps were aligned and displayed with the MapSolver 3.1 software. Aligned regions are in blue. Insertions relative to a genome are in white. Inversions and duplications are in red. Click here for file
