# Supplementary figures and images for: Staphylococcus epidermidis pan-genome sequence analysis reveals diversity of skin commensal and hospital infection-associated isolates
Source: Genome Biol. 2012 Jul 25;13(7):R64. doi: 10.1186/gb-2012-13-7-r64 (PMC4053731; doi:10.1186/gb-2012-13-7-r64)

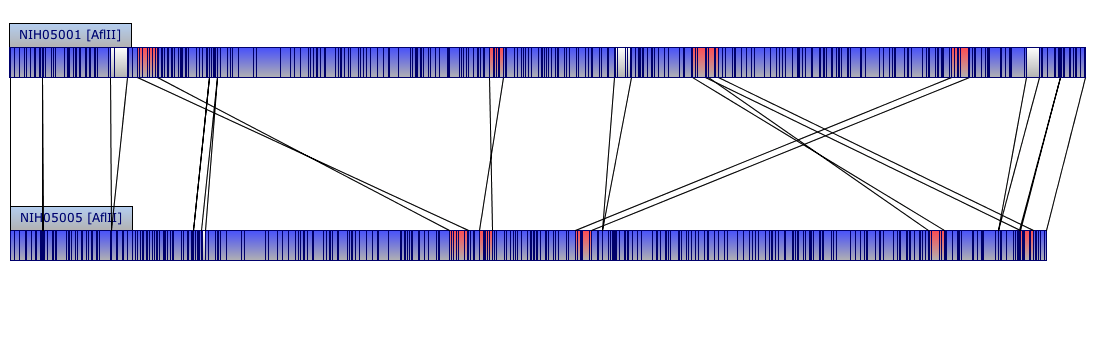

Supplement: Additional file 3 — Optical mapping of ST2 isolates NIH05001 and NIH05005. Optical maps were aligned and displayed with the MapSolver 3.1 software. Aligned regions are in blue. Insertions relative to a genome are in white. Inversions and duplications are in red. [file gb-2012-13-7-r64-S3.PNG]
